# Diminished Anticipatory and Consummatory Pleasure in Dysphoria: Evidence From an Experience Sampling Study

**DOI:** 10.3389/fpsyg.2019.02124

**Published:** 2019-09-19

**Authors:** Xu Li, Yu-Ting Zhang, Zhi-Jing Huang, Xue-Lei Chen, Feng-Hui Yuan, Xiao-Jun Sun

**Affiliations:** ^1^School of Psychology, Central China Normal University, Wuhan, China; ^2^Key Laboratory of Adolescent Cyberpsychology and Behavior, Ministry of Education, Central China Normal University, Wuhan, China; ^3^School of Sociology, Central China Normal University, Wuhan, China

**Keywords:** anhedonia, anticipatory pleasure, consummatory pleasure, experience sampling, dysphoria

## Abstract

Anhedonia, the experience of diminished pleasure, is a core feature of major depressive disorder and is often present long before the diagnosis of depression. Most previous studies have investigated anhedonia with self-report measures of trait anhedonia or with behavioral paradigms using laboratory stimuli, and the real-time characteristics of hedonic processing in subclinical depression remain under-investigated. We used the experience sampling method to evaluate momentary experience of hedonic feelings in the context of daily life. Dysphoric (*n* = 49) and non-dysphoric (*n* = 51) college students completed assessments of their current positive affect (PA), as well as state anticipatory and consummatory pleasure, 3 or 4 times a day every day for 2 weeks. The results showed that dysphoric individuals reported less state anticipatory and consummatory pleasure compared with non-dysphoric individuals. Moreover, significant time-lagged associations between anticipatory pleasure and follow-up consummatory pleasure were found in the whole sample, after adjustment for current PA. The current findings thus hold considerable promise in advancing our understanding of anhedonia as well as the important role of state anticipatory pleasure in relation to depression.

## Introduction

Anhedonia, the diminished ability to experience pleasure, manifests as a transdiagnostic symptom among individuals with different psychiatric disorders (Thomsen et al., 2015). For this reason it has been identified by the Research Domain Criteria (RDoC) initiative as key to the investigation of behavioral and clinical symptoms across disorders (Insel et al., 2010). In the case of major depressive disorder (MDD), anhedonia is an essential diagnostic feature, and the severity of anhedonia has been found to correlate with the severity of depressive symptoms and time to remission (McMakin et al., 2012).

However, accumulating evidence suggests that anhedonia is not a unitary construct. Two independent sub-components of the hedonic processing function have been revealed: anticipatory pleasure and consummatory pleasure (Kring and Caponigro, 2010; Treadway and Zald, 2011). Anticipatory pleasure refers to pleasure derived from predicted future events; in contrast, consummatory pleasure involves the experience of pleasure during current events. The importance of such a distinction has been clear in other disorders. For example, anhedonia in schizophrenia has been demonstrated in anticipatory but not consummatory pleasure, in patients as well as in individuals at risk for schizophrenia (Li et al., 2015). More research is warranted to investigate the distinct roles of anticipatory and consummatory pleasure and their contributions to the pathology seen in depression.

Although it is well-documented that anhedonia plays a role in depression, the role of each type of anhedonia is not well-understood. Most empirical studies on anhedonia in depression have primarily focused on consummatory pleasure. Depressed individuals have been shown to report reduced emotional reactivity to stimuli used in laboratory research (Bylsma et al., 2008), and to demonstrate lower positive reactivity and elevated negative reactivity toward daily events (Bylsma et al., 2011). In addition, significant aberrations of brain function related to consummatory pleasure have been reported in individuals at risk for depression (Foti et al., 2011; Bress et al., 2012). In these studies reward-related brain areas such as the caudate, putamen, and anterior cingulate cortex were less activated in individuals with depression. Such blunted reactivity has also been detected in individuals at risk for depression (Saxena et al., 2017), suggesting that depression is robustly associated with reduced behavioral and neural hedonic responsivity, i.e., reduced consummatory pleasure.

Compared to consummatory pleasure, less is known about anticipatory pleasure in depression. A few studies have investigated anticipatory and consummatory pleasure simultaneously, and these studies have produced mixed findings. In an MDD sample, Sherdell et al. (2012) observed no deficits in reward seeking behavior or in consummatory responses to rewards in a laboratory based effort-reward task, suggesting intact anticipatory and consummatory hedonic processing capacity in depression. However, studies utilizing self-report measures of anhedonia have shown both blunted anticipatory and blunted consummatory pleasure in depression (Liu et al., 2012; Sherdell et al., 2012; Yang et al., 2014). Moreover, based on self-report, diminished anticipatory pleasure in depression, rather than consummatory pleasure, was found to be a significant predictor of effort expended for future rewards (Sherdell et al., 2012). Hence, both intact and reduced hedonic capacity have been found to relate to depression. One possible explanation for the differing results may have to do with the operationalization of anhedonia. Experimental studies evaluated dynamic hedonic responses on a trial-by-trial basis with laboratory-based task, while more static estimates of hedonic capacity were obtained with self-report measures.

Importantly, however, both the experimental method and self-report method have limitations in the study of anhedonia. On one hand, stimuli used in experimental studies (e.g., monetary rewards, positive images) are often standardized to obtain more precise control of confounding factors, but because contextual information is often removed or obscured, laboratory tasks might not be analogous to the actual changing contexts of everyday life. On the other hand, in self-report studies, participants are indicated to rate their pleasure experience in response to a hypothetical situation rather than actual situation. The self-report measures of hedonic experience rely heavily on retrospective recall and therefore scores derived from these measures might be confounded by the severity of memory deficits of the clinical sample characterized by anhedonia (Liu et al., 2012; Olsen et al., 2015). The reduced recall accuracy for specific personal memories has been suggested to be associated with reduced expectations for future events (Schacter et al., 2008). More critically, memory bias toward mood-congruent negative content is strongly related to depressive symptomatology and represents a primary mechanism in depression (Marchetti et al., 2018). Thus, retrospective reports might not provide a comprehensive representation of the fluctuating emotional experiences in daily life.

The experience sampling method (ESM) is a technique that enables real-time and repeated sampling, which could yield more reliable and accurate estimates than single-point assessment or retrospective reports (Csikszentmihalyi and Larson, 1987). Given the fact that emotional feelings are highly variable, the ESM is now generally considered as the “gold standard” to capture the dynamical signature of subjective experience (Shiffman et al., 2008). Important for the current study, the ESM has been frequently used to examine the daily fluctuations of affect in depression, with results showing that depression involves alteration in the mean level of both positive affect (PA) and negative affect (Kuppens et al., 2010; Thompson et al., 2012). The ESM makes it possible to examine how pleasure unfold in the context of daily life and thus offers insights into factors affect pathogenic processes and pathological states of depression.

Studies have started to employ ESM to investigate the temporal features of anticipatory and consummatory pleasure in the context of daily life. The first study was conducted in schizophrenia by Gard et al. (2007), in which deficits in anticipatory but not consummatory pleasure were reported, while recent ESM studies suggested intact capacity to experience pleasure during reward anticipation in schizophrenia (Gard et al., 2014; Edwards et al., 2018). However, the distinctive momentary feature of anticipatory and consummatory pleasure in relation to depression has rarely been studied. In a recent ESM study (Heininga et al., 2019), temporal consummatory pleasure of anhedonic individuals with current MDD episode was examined, the results showed that the frequency of reward experience in anhedonic individuals with current MDD episode were at equivalent levels as in healthy controls, suggesting intact consummatory pleasure in MDD with anhedonic symptoms. The first ESM study (Wu et al., 2017) investigating both daily anticipatory and consummatory pleasure of MDD, however, demonstrated that MDD patients reported blunted anticipatory and consummatory pleasure for daily activities. Although the accuracy of anticipatory ratings for pleasure could be estimated by subtracting consummatory ratings from anticipatory ratings in the Wu et al. (2017) study, this practice could not take full advantage of a key strength of ESM, which is the ability to capture the interactions between the two components of anhedonia. Cross-lagged regression models, on the other hand, could make use of the time-lagged structure of the data and thus could provide a better estimate of the moment-to-moment interplay between anticipatory ratings and consummatory ratings. The time-lagged analysis was utilized in present study to examine the extent to which anticipatory pleasure is related to in-the-moment experience of pleasure in the flow of daily life.

Although previous studies have provided preliminary evidence that patients with MDD had reduced daily experience of pleasure, it remains unknown whether these dysfunctions of hedonic processing emerge in dysphoria, who demonstrate elevated levels of depressive symptoms, but do not meet the diagnostic criteria for MDD. Dysphoric individuals showed impairment in cognitive function, such as working memory and interference control (Owens et al., 2012); and they reported more frequent use of non-adaptive emotional regulation strategies and experienced more negative affect than non-dysphoric individuals (Quigley and Dobson, 2014). Dysphoric individual are found to be at increased risk for developing MDD in the future (Gotlib et al., 1995), especially when dysphoria is present during young adulthood (Wilcox and Anthony, 2004). A recent ESM study provided preliminary evidence for blunted consummatory pleasure in individuals with anhedonia (Heininga et al., 2017). The temporal unfolding of anticipatory pleasure and the predictive relationship between anticipatory and consummatory pleasure in dysphoria, however, have not been fully studied. Given the close relationship between dysphoria and depression, we speculate that diminished anticipatory and consummatory pleasure might be observed in people with dysphoria.

In the affect literature, pleasure has been considered a dimension of PA. Research investigating the characteristics of PA in depression has suggested that depressive symptoms are related to lower PA both in clinical (Thompson et al., 2012) and non-clinical samples (van Roekel et al., 2016), while the functioning of PA in anhedonia is not yet fully understood. Most experimental studies have examined pleasure ratings in response to stimuli without considering the affective context, especially PA. The dynamic interaction of PA, negative affect and stress experience differed between anhedonic and non-anhedonic individuals with subclinical depression (Bos et al., 2018), suggesting an interplay between anhedonia and temporal affective experience. Our study extends the prior research by considering the impact of PA on the anticipatory and consummatory components of the experience of pleasure in dysphoria.

The primary goal of the current study was to understand the nature of anticipatory and consummatory pleasure in individuals with dysphoria in college-age population by using ESM. By studying individuals at risk for depression, mechanisms that underlie the future development of MDD episode may be uncovered. Furthermore, the recruitment of dysphoric individuals could overcome some of the confounding factors when studying patients with MDD, such as medication, severity of illness and episode number. MDD has its peak incidence in young adults (Kessler et al., 2003), thus a focus of college-age sample is of great relevance. Trait dispositions in anticipatory and consummatory pleasure were assessed with questionnaires. Other data were collected using ESM. Specifically, all participants provided ratings of temporal PA, anticipatory pleasure, and consummatory pleasure in response to phone prompts 3 to 4 times a day for 14 days. It was hypothesized that dysphoric individuals would report less PA, as well as less state anticipatory and consummatory pleasure. Moreover, we explored the potential role of anticipation of pleasure in predicting current pleasure, and whether this relationship varied between individuals with and without dysphoria. Because few studies have examined the predictive relationship between anticipatory and consummatory pleasure, no hypothesis was made regarding group difference, and the relationship between anticipatory pleasure and current pleasure was examined separately in each group. This study also examined the extent to which PA influences the experience of pleasure, and whether PA changes the relationship between anticipatory pleasure and consummatory pleasure. Finally, exploratory analyses of group differences on these time-lagged associations between dysphoric and non-dysphoric groups were performed.

## Materials and Methods

### Participants

Three hundred and forty-one college students from Wuhan, China were invited to complete the Beck Depression Inventory-II (BDI-II, Beck et al., 1996), which taps into the severity of depressive symptomology in the general population. The BDI-II is currently one of the most widely used measure for assessing depression, and it has been shown to have good sensitivity and specificity in screening MDD in college-student population (Shean and Baldwin, 2008). Participants who scored 20 (moderate depression) or higher were identified as dysphoric individuals, and participants who scored lower than 14 (minimal depression) were classified as non-dysphoric controls, the BDI-II cut-off for dysphoric and non-dysphoric individuals was consistent with previous studies with college-student sample (Lissnyder and Koster, 2010; Owens et al., 2012; Quigley and Dobson, 2014). Potential participants who met either of these cut-off criteria on the BDI-II were screened by phone to check for availability to participate in the experience sampling study. Based on the phone interview, individuals with a history of psychiatric disorders, brain injury, or substance abuse were not included. As a result, 51 dysphoric individuals and 56 non-dysphoric controls were included in the present study. After baseline assessment, two participants from the dysphoric group and three from the non-dysphoric control group dropped out due to scheduling problems.

This study was approved by the Ethics Committee of the Central China Normal University. Written informed consent was obtained from all participants at the beginning of the baseline assessment. Participants were told they could stop participating at any time without penalty. At the end of the study participants received compensation of 30 RMB.

### Baseline Assessment

On the first day they arrived at our laboratory, participants were administered two self-report measures. The Temporal Experience of Pleasure Scale (TEPS, Gard et al., 2006) is a self-report measure that assesses the trait dispositions of anticipatory pleasure and consummatory pleasure. The Chinese version of the TEPS (Chan et al., 2012) contains 20 items and participants answered each item on a 6-point Likert scale ranging from 1 (“very false for me”) to 6 (“very true for me”). Higher scores indicate greater hedonic capacity. Cronbach’s α was 0.759 for the whole scale, 0.726 for the consummatory pleasure subscale, and 0.530 for the anticipatory pleasure subscale. The Snaith–Hamilton Pleasure Scale (SHAPS), developed by Snaith et al. (1995), is a self-rating scale used to measure trait consummatory pleasure in certain situations. Each item is rated on a four-point Likert scale from 1 (“definitely agree”) to 4 (“definitely disagree”). The SHAPS contains 14 items, and higher scores indicate more severe deficits in consummatory pleasure. The Chinese version of the SHAPS has good internal consistency (Liu et al., 2012). Cronbach’s α in the present study was 0.891.

### ESM Procedure

An Internet link containing the set of ESM assessments was sent to each participant’s smart phone 3–4 times each day between 8:00 a.m. to 10:00 p.m., with a time interval of more than 90 min between two adjacent points, for 14 consecutive days. This resulted in a maximum of 56 events per person. Participants were asked to complete the brief assessments as soon as they received the link on their smartphone. If no response was given within 30 min or the participant spent more than 180 s to complete the assessments, the data were considered missing. Two participants from the non-dysphoric group were removed from the final analysis because their response rates were lower than 50%. Thus the final sample consisted of 49 participants in the dysphoric group and 51 participants in the non-dysphoric group. No significant group differences were observed in gender, age, or education (Table 1).

**TABLE 1:** Sample characteristics.

	**Dysphoric group (*n* = 49)**	**Non-dysphoric group (*n* = 51)**	***t/χ2***	***p***
			
	**(*M* ± *SD*)**	**(*M* ± *SD*)**		
Age	20.22 ± 1.37	20.78 ± 1.74	1.79	0.08
Gender (% female)	85.71	82.35	0.21	0.79
Education (years)	14.20 ± 1.14	14.75 ± 1.56	1.99	0.50
BDI-II	24.78 ± 4.91	7.73 ± 3.67	19.72	<0.01
TEPS_total	82.39 ± 12.86	85.73 ± 9.29	1.49	0.14
Anticipatory pleasure	37.45 ± 6.64	39.12 ± 4.43	1.47	0.15
Consummatory pleasure	44.94 ± 7.61	46.61 ± 6.55	1.18	0.24
SHAPS	21.47 ± 6.32	20.73 ± 5.17	0.65	0.52

*BDI-II, Beck Depression Inventory-II; TEPS, The Temporal Experience of Pleasure Scale; SHAPS, The Snaith–Hamilton Pleasure Scale.*

#### Momentary PA

Momentary PA was measured with the 10 positive items (alert, excited, enthusiastic, attentive, proud, inspired, interested, determined, strong, active) from the Positive and Negative Affect Schedule (PANAS; Watson et al., 1988) using the structure “At this moment I feel…” For each item, participants were asked to indicate the extent to which they were experiencing a certain affect at that moment, from 1 (“not at all”) to 5 (“very much”).

#### State Anticipatory Pleasure and Consummatory Pleasure

To assess consummatory pleasure, participants firstly were asked to indicate what they were doing at that moment, by selecting from several options (i.e., work/study, sleeping, eating, daydreaming, cleaning, playing video games, interacting on social media, shopping, watching movies, going on a date, group discussion, other) and then to rate the amount of pleasure they were experiencing from 1 (“none”) to 10 (“an extremely large amount”) on a visual analog scale. To measure anticipatory pleasure at the state level, participants were asked to indicate what kind of activity they would be involved in next, and then they were asked to forecast the amount of enjoyment they would derive from that activity from 1 (“none”) to 10 (“an extremely large amount”). By including questions regarding current and future activities, the design helps to ensure that ratings for consummatory pleasure and anticipatory pleasure were based on daily events rather than memory representations. The use of brief measures is common in ESM research because excessive length can compromise response quality and reduce compliance (Bakker et al., 2017; Starr and Hershenberg, 2017; Edwards et al., 2018).

### Data Analysis

Group differences on demographic information and baseline self-report measures were analyzed with SPSS 23.0. The ESM data have a hierarchical structure in which within-person daily observations (Level 1; e.g., state experience of pleasure) are nested within between-person characteristics (Level 2; e.g., dysphoric status). Therefore, data were analyzed by means of multilevel modeling in Mplus version 7.4. Separate models were estimated for daily PA, state anticipatory pleasure, and state consummatory pleasure. Furthermore, all Level 1 predictors were person-mean centered (i.e., around each participant’s mean score) to separate within-person effects from between-person effects (Finch and Bolin, 2017, pp 38–39). Intercepts and slopes of level 1 were modeled as random effects, allowing the intercepts and slopes to vary at between-person level (i.e., level 2) (Nezlek, 2012).

Before running models to test our hypotheses, we first ran null models in Mplus (i.e., containing no predictors at any level) with PA, state anticipatory pleasure and state consummatory pleasure as the outcome variables. This type of analysis can be used to calculate intraclass correlation coefficients (ICCs), which provides an estimate of the proportion of variance in the outcome variable accounted for by the between-person level, which reflects individual differences (vs. the within-person level, which reflects situational differences).

#### Associations Between Momentary Pleasure and Dysphoric Status

To examine whether dysphoric status predicted differences in anticipatory and consummatory pleasure across daily observations, we regressed the within-person parameters on dysphoric status (i.e., 0 = non-dysphoric group; 1 = dysphoric group) (see section “Model 1”).

### Model 1

Level 1: PA_*ij*_ or anticipatory pleasure _*ij*_ or consummatory pleasure _*ij*_ = β_0*j*_ + r_*ij*_

Level 2: β_0*j*_ = γ_00_+ γ_01_ (dysphoric status _*j*_) + U_0*j*_

In the equations, *i* represents time point, and *j* represents person. The outcome at Level 1 (e.g., PA), representing the observed score of person *j*’s at time *i*, was modeled as a function of a random intercept (β_0*j*_) representing the within-person mean of the corresponding outcome variable, r_*ij*_ represents residual errors at within-person level. At Level-2, γ_00_ represents mean pleasure for the non-dysphoric group, and γ_01_ represents the difference in mean pleasure between the dysphoric and non-dysphoric groups. U_0*j*_ represents residual errors at between-person level.

Next, to reveal the unique associations between momentary pleasure and dysphoria, we reran the full models, controlling for PA (see section “Model 2”).

### Model 2

Level 1: consummatory pleasure _*ij*_ or anticipatory pleasure _*ij*_ = β_0*j*_ + β_1*j*_ PA_*ij*_ + r_*ij*_

Level 2: β_0*j*_ = γ_00_+ γ_01_ (dysphoric status _*j*_) + U_0*j*_

β_1*j*_ = γ_10_+ γ_11_ (dysphoric status _*j*_) + U_1*j*_

The outcome at Level 1 (e.g., consummatory pleasure), representing the observed score of person *j*’s score at time *i*, was modeled as a function of a random intercept (β_0*j*_) representing the mean pleasure of person *j*, and a random slope (β_1*j*_) representing the relationship between a person *j*’s PA at time *t* and consummatory pleasure at time *t.*γ_00_ and γ_10_ represent the average (or fixed-effects) estimates of consummatory pleasure and PA of the non-dysphoric group, while γ_01_ and γ_11_ represent group differences of consummatory pleasure and PA between the dysphoric and non-dysphoric groups.

#### Concordance Between Anticipatory and Consummatory Pleasure

Finally, to investigate how much the anticipated pleasure for an activity is concordant with the amount of consummatory pleasure, time-lagged associations between anticipatory pleasure and consummatory pleasure were estimated with an autocorrelation approach. In particular, we conducted multilevel modeling to predict consummatory pleasure at sampling moment *t* from anticipatory pleasure at *t*-1 (see section “Model 3”). Additionally, consummatory pleasure at *t*-1 and PA at *t* were included as covariates separately (see sections “Model 4, 5”) and simultaneously (see section “Model 6”) to control for potential confounding effects. Moreover, to examine whether dysphoria moderate these associations, a group variable indicating dysphoric status was added as the Level 2 predictor to test for group differences between the dysphoric and non-dysphoric groups (see Supplementary Materials for Model equations and further details).

### Model 3

Level 1: consummatory pleasure _*ij*_ = β_0*j*_ + β_1*j*_ (anticipatory pleasure _*t*–1_) + r_*ij*_

Level 2: β_0*j*_ = γ_00_ + U_0*j*_

β_1*j*_ = γ_10_ + U_1*j*_

### Model 4

Level 1: consummatory pleasure _*ij*_ = β_0*j*_ + β_1*j*_ (anticipatory pleasure _*t*–1_) + β_2*j*_ (consummatory pleasure _*t*–1_) + r_*ij*_

Level 2: β_0*j*_ = γ_00_ + U_0*j*_

β_1*j*_ = γ_10_ + U_1*j*_

β_2*j*_ = γ_20_ + U_2*j*_

### Model 5

Level 1: consummatory pleasure _*ij*_ = β_0*j*_ + β_1*j*_ (anticipatory pleasure _*t*–1_) + β_2*j*_ (PA _*t*_) + r_*ij*_

Level 2: β_0*j*_ = γ_00_ + U_0*j*_

β_1*j*_ = γ_10_ + U_1*j*_

β_2*j*_ = γ_20_ + U_2*j*_

### Model 6

Level 1: consummatory pleasure _*ij*_ = β_0*j*_ + β_1*j*_ (anticipatory pleasure _*t*–1_) + β_2*j*_ (consummatory pleasure _*t*–1_) + β_3*j*_ (PA _*t*_) + r_*ij*_

Level 2: β_0*j*_ = γ_00_ + U_0*j*_

β_1*j*_ = γ_10_ + U_1*j*_

β_2*j*_ = γ_20_ + U_2*j*_

β_3*j*_ = γ_30_ + U_3*j*_

In the equations, the outcome at Level 1 (consummatory pleasure_*ij*_), representing a person *j*’s score on consummatory pleasure items at time *i*, was modeled as a function of a random intercept (β_0*j*_) and random slopes (β_1*j*_, β_2*j*_, β_3*j*_). β_0*j*_ represents person *j*’s mean score for consummatory pleasure *t*; β_1*j*_ represents the relationship between a person *j*’s anticipatory pleasure at time *t*-1 and consummatory pleasure at time *t*; β_2*j*_ represents the relationship between person *j*’s current consummatory pleasure and their consummatory pleasure at the prior time point in Model 4 (current PA in Model 5). β_3*j*_ represents the relationship between person *j*’s current consummatory pleasure and current PA. r_*ij*_ represents residual errors at within-person level.

The Level-2 parameter estimates (γ_00_, γ_10_, γ_20_, γ_30_) represent the average (or fixed-effects) estimates across participants. U_0*j*_, U_1*j*_, U_2*j*_, and U_3*j*_ represent residual errors at between-person level.

## Results

### Self-Report Measures

There were no significant group differences between the dysphoric and non-dysphoric group on the TEPS total score, *t*(98) = 1.49, *p* = 0.14, anticipatory pleasure subscale, *t*(83.25) = 1.47, *p* = 0.15, or consummatory pleasure subscale, *t*(98) = 1.18, *p* = 0.24. Moreover, no significant group difference was observed on the SHAPS total score, *t*(98) = 0.65, *p* = 0.52. These results suggested that the dysphoric group demonstrated no attenuation of trait anticipatory or consummatory pleasure.

### ESM Estimates of State Anticipatory and Consummatory Pleasure

To determine whether age, gender, education, and time of assessments covaried with our dependent variables, we regressed these variables on consummatory pleasure_*ij*_, anticipatory pleasure*_ij_*, and PA_*ij*_ separately. Results showed that those dependent variables did not vary by age, gender, education, or time. Thus, these demographic variables and time were not included as covariates in further analyses.

The ICCs from the null models revealed that 39.9% of the variance in PA, 34.9% of the variance in state anticipatory pleasure and 39.4% of the variance in state consummatory pleasure was at the between-person level, suggesting that both the temporal situation and dysphoric status play a role in momentary PA and the experience of pleasure in daily life. Several multilevel models were tested to examine the distinct contribution of dysphoric status to momentary pleasure respectively.

No significant group difference was observed in PA between the dysphoric and non-dysphoric groups (*p* = 0.64). However, individuals in the dysphoric group reported lower levels of state anticipatory pleasure (β = −0.77, *SE* = 0.23, *p* < 0.01) and consummatory pleasure (β = −0.82, *SE* = 0.23, *p* < 0.01) than individuals in the non-dysphoric group. Moreover, group differences between the dysphoric and non-dysphoric group in state anticipatory and consummatory pleasure remained statistically significant after including PA as a covariate (Table 2).

**TABLE 2:** Estimates of positive affect (PA), anticipatory and consummatory pleasure in individuals with dysphoria compared with non-dysphoric individuals.

	**Outcome variable**	**Covariate**	**β**	***SE***	***p***
**Model 1**					
	PA	-	0.13	0.29	0.64
	Anticipatory pleasure	-	–0.77	0.23	< 0.01
	Consummatory pleasure	-	–0.82	0.23	< 0.01
**Model 2**					
	Anticipatory pleasure		–0.77	0.23	< 0.01
		PA	0.18	0.19	0.35
	Consummatory pleasure		–0.82	0.23	< 0.01
		PA	0.36	0.20	0.08

### Impact of Anticipatory Pleasure on Consummatory Pleasure

Results of tests of Models 3–6, namely time-lagged analyses examining associations between anticipatory pleasure and changes in consummatory pleasure in the dysphoric and non-dysphoric groups separately, are presented in Table 3. Model 3 tested consummatory pleasure at the current moment as a function of anticipated pleasure at the prior time point. We found that greater anticipatory pleasure was a significant predictor of greater follow-up consummatory pleasure in the dysphoric group (β = 0.15, *SE* = 0.04, *p* < 0.01), but not the non-dysphoric group (β = 0.05, *SE* = 0.04, *p* = 0.22). In addition, the significant association between anticipatory pleasure at *t*-1 and consummatory at *t* in the dysphoric group remained unchanged after controlling for consummatory pleasure _*t*–1_ (Model 4), PA _*t*_ (Model 5), or both of them (Model 6), all *ps* < 0.01. In the non-dysphoric group, no significant relationship was found between anticipatory pleasure and consummatory pleasure, all *p*s > 0.05.

**TABLE 3:** Multilevel analyses of the time-lagged associations between consummatory pleasure, anticipatory pleasure and positive affect (PA) in dysphoric and non-dysphoric groups respectively and as a whole sample.

**Outcome variable:**	**Predictors**	**Dysphoric group**		**Non-dysphoric group**		**The whole sample**	
				
**Consummatory pleasure (*t*)**		***β(SE)***	***p***	***β(SE)***	***p***	***β(SE)***	***p***
**Model 3**							
	Anticipatory pleasure (*t*-1)	0.15 (0.04)	< 0.01	0.05 (0.04)	0.22	0.08 (0.04)	0.07
**Model 4**							
	Anticipatory pleasure (*t*-1)	0.12 (0.03)	< 0.01	0.03 (0.03)	0.30	0.05 (0.04)	0.14
	Consummatory pleasure (*t*-1)	0.06 (0.04)	0.11	0.07 (0.03)	0.03	0.08 (0.03)	< 0.01
**Model 5**							
	Anticipatory pleasure (*t*-1)	0.08 (0.02)	< 0.01	0.03 (0.02)	0.09	0.04 (0.02)	0.02
	PA (*t*)	1.83 (0.15)	< 0.01	1.65 (0.13)	< 0.01	1.81 (0.10)	< 0.01
**Model 6**							
	Anticipatory pleasure (*t*-1)	0.05 (0.02)	< 0.01	0.03 (0.02)	0.10	0.03 (0.02)	0.04
	Consummatory pleasure (*t*-1)	0.04 (0.03)	0.24	0.03 (0.03)	0.23	0.04 (0.02)	0.07
	PA (*t*)	1.89 (0.14)	< 0.01	1.72 (0.13)	< 0.01	1.80 (0.10)	< 0.01

The moderating effects of dysphoria on these associations were not significant (all *p*s > 0.05, see Supplementary Table S1). Therefore, we rerun the models 3–6 by including all participants to determine if the predictive relationships between anticipatory pleasure and consummatory pleasure were statistically significant in the whole sample. The results showed that anticipatory pleasure alone positively predict current levels of consummatory pleasure at the trend level, β = 0.08, *SE* = 0.04, *p* = 0.07; after controlling for current PA, a significant association was found between anticipatory pleasure at *t*-1 and consummatory at *t*, β = 0.04, *SE* = 0.02, *p* = 0.02. In addition, the association between current PA and consummatory pleasure was also significant, *p* < 0.01, indicating that a higher level of anticipatory pleasure at prior time point in conjunction with higher current PA are associated with greater experience of consummatory pleasure for all participants.

## Discussion

The aim of this study was to investigate state anhedonia in subclinical depression, taking into account trait anhedonia and current PA. ESM was utilized to track the momentary experience of anticipatory and consummatory pleasure over the course of 14 consecutive days in dysphoric and non-dysphoric individuals. Multilevel analyses showed that compared to the non-dysphoric individuals, the dysphoric individuals showed less pleasure during the anticipation of future events and the engagement of on-going activities, and this pattern remained unchanged after adding temporal PA as a covariate. However, no group differences between dysphoric and non-dysphoric individuals were observed regarding trait anhedonia. Moreover, a significant predictive relationship between state anticipatory and consummatory pleasure after adjustment for temporal PA was revealed in the whole sample. Investigating the distinct nature of anticipatory and consummatory pleasure in daily life may help us to understand the mixed findings of prior studies on anhedonia in depression.

Consistent with our hypothesis, dysphoric individuals, compared to non-dysphoric individuals, reported lower levels of pleasure during anticipation of an upcoming event. This finding is consistent with experimental studies investigating anticipatory responses to laboratory stimuli in MDD (McFarland and Klein, 2009; Admon and Pizzagalli, 2015) and in dysphoria (Yuan and Kring, 2009). For example, in the study of McFarland and Klein (2009), compared to healthy controls, patients with MDD reported less PA when anticipating monetary reward. Similarly, neuroimaging studies have found blunted response during anticipation at the ventral striatum, a core region involved in reward processing, both in patients with MDD (Arrondo et al., 2015; Keren et al., 2018) and in individuals at high risk for depression (Olino et al., 2014), suggesting dysfunctional anticipatory processing in response to laboratory stimuli. The key finding that dysphoric individuals report less pleasure when anticipating future events is also consistent with research on depressed individuals’ forecasts of the intensity of their PA. In general, people are over-optimistic about their future, with evidence showing that people have a tendency to overestimate the intensity and duration of PA than that they actually experience (Wenze et al., 2012; Morewedge and Buechel, 2013). This unrealistic prediction about emotional experience, though not accurate, is beneficial in maintaining mental health. With regard to evidence for biased predictions of PA in relation to depression, more severe depressive symptoms have been shown to be associated with less optimistic bias in PA prediction on a daily and a weekly basis both in the general population (Hoerger et al., 2012; Wenze et al., 2012, 2013) and in individuals with clinical and subclinical depression (MacLeod and Salaminiou, 2001; Chentsova-Dutton and Hanley, 2010). The same pattern of reduced anticipated PA has also been found in patients with remitted MDD (Thompson et al., 2017), suggesting that reduced anticipated positive experience might represent a trait-like marker in depression.

Only recently have researchers directly investigated depressed persons’ anticipatory pleasure using ESM. Greater severity of subclinical symptoms of depression in the general population are found to be predictive of reduced pleasure during anticipating positive events in daily life (Bakker et al., 2017; Starr and Hershenberg, 2017). Our findings are also consistent with Wu et al.’s (2017) study in which patients with MDD demonstrated blunted state anticipatory and consummatory pleasure in relation to daily activities. On the other hand, Wu et al. (2017) also showed that patients with MDD reported levels of trait anticipatory and consummatory pleasure that were comparable to those reported by healthy controls. Self-report measures of trait anhedonia have been criticized for assessing responses to hypothetical situations that may not be comparable to actual situations, suggesting the importance of using multiple measures of anhedonia in any given study. ESM appears to be more sensitive than other methods of measuring the hedonic function of dysphoria. Given the transdiagnostic nature of anhedonia and its importance in depression, deficits in state anticipatory and consummatory pleasure might represent a core dysfunction of hedonic processing in people with dysphoria.

Daily PA is typically measured in general terms, with only limited research on the temporal experience of pleasure in response to daily events. Our finding that dysphoric and non-dysphoric individuals did not differ in their mean level of PA is consistent with study of subclinical samples (Olino et al., 2014). Moreover, in our study, dysphoric individuals, compared to non-dysphoric individuals, reported less consummatory pleasure in the course of the day. Consistent with our findings, previous research showed that patients with MDD are less responsive to positive stimuli in a laboratory task (Bylsma et al., 2008). Research with patients suffering from schizophrenia has also demonstrated a dissociation between PA and consummatory pleasure, with intact PA function but altered hedonic experience in patients compared to healthy controls (Edwards et al., 2018). In a study designed to distinguish PA and consummatory pleasure, Heininga et al. (2017) found that individuals with anhedonia reported a lower level of both PA and consummatory pleasure compared to individuals without anhedonia. Future research should further investigate the roles of the temporal experience of pleasure and current PA in depression, as well as their distinct contributions to the pathology of depression.

With regard to the predictive relationship between anticipatory and consummatory pleasure, separate models conducted within the two groups revealed significant associations between anticipatory pleasure and consummatory pleasure in the dysphoric group but not non-dysphoric group. However, models including dysphoria status as predictor did not support the moderating effect of dysphoria on these associations. Anticipatory pleasure was a significant predictor of consummatory pleasure in the whole sample, after adjustment for current PA. Thus, dysphoric and non-dysphoric individuals both report higher levels of consummatory pleasure when they anticipated greater pleasure at the previous time point, although dysphoria is associated with lower levels of anticipatory and consummatory pleasure. This result is in line with the ESM study using end-of-day dairy reports, in which it was found that the increased anticipation for positive experience and positive events of the next day predicted greater reduction of depressed symptoms in dysphoria (Starr and Hershenberg, 2017).

Levels of anticipatory pleasure are predictors of effort-expenditure for rewards in healthy volunteers (Geaney et al., 2015), and it has been suggested that depression related anticipatory pleasure deficiency is associated with impairment in translating motivation to rewarding activities (Sherdell et al., 2012; van Roekel et al., 2016; Bakker et al., 2017). For example, Bakker et al. (2017) found that the more severe the depressive symptoms, the less likely that increases in reward anticipation are to be followed by increases in PA. Moreover, the positive relationship between reward anticipation and activity engagement in controls was also diminished in individuals with dysphoria. This suggests that dysphoric individuals were not able to translate their anticipation into goal-directed behaviors to pursue the anticipated reward, which might further prevent them from experiencing pleasure. The assumption of the mediating role of approach motivation in the translational mechanism from anticipatory pleasure to the experience of consummatory pleasure remains to be tested directly in laboratory and naturalistic settings. Although further validation is required, the predictive relationships between anticipatory and consummatory pleasure in dysphoria suggest that the dysfunctional reward anticipation represents a key component of anhedonia in relation to depression.

Together, these results suggest that the lack of anticipatory pleasure may be a dominant component of impairment that affects motivated behavior and subsequent experiential feelings in depression, and deficits in anticipatory pleasure might represent a critical therapeutic target requiring further investigation. Previous studies have provided preliminary evidence for the efficacy of interventions aimed at increasing anticipatory pleasure, schizophrenia patients reported higher levels of consummatory pleasure and more frequent engagement in daily activities at the end of training (Favrod et al., 2010; Nguyen et al., 2016). Future research could examine the extent to which benefits from interventions designed to improve depressed individuals’ anticipation of pleasure could translate into downstream enjoyment of pleasurable experiences.

### Limitations

Our study has several limitations. Firstly, a disproportionate number of female participants were recruited, thus our results cannot be generalized to other population. Secondly, the estimated internal consistency of anticipatory subscale of TEPS is not as strong as that of consummatory subscale in present study. Anticipatory pleasure ratings are more likely to be affected by the context and therefore show less consistency than consummatory pleasure ratings (Edwards et al., 2015). In addition, the high homogeneity of college-student participants would reduce the variability in measurements (Peterson, 2001) and may affect score reliability. Thirdly, whether there are long-lasting and additive effects of anticipatory pleasure on consummatory pleasure will need to be established by further research, given evidence that anticipatory stressor together with current stressor predicted higher levels of negative affect 2.5 h later (Scott et al., 2018). Additional limitations to this study concern methodological problems in modeling time-lagged relationships, i.e., time-interval dependency (Kuiper and Ryan, 2018). Specifically, the event at *t* might not be exactly the one predicted at *t*-1, and thus the predictive relationship between anticipatory and consummatory pleasure might not reliably assess hedonic responses to the same events. To gain a better understanding of this correspondence, further research could increase the sampling density and include a more objective measure of the context in which individuals rate their experiences (e.g., by having participants indicate whether the current activity is the one that at the previous assessment they expected to be engaged in).

## Conclusion

This study makes an important contribution to the literature of anhedonia in depression by investigating the momentary level of anticipatory and consummatory pleasure during daily life activities in dysphoric and non-dysphoric individuals. The results showed that dysphoric individuals were characterized with less state anticipatory and consummatory pleasure compared with non-dysphoric individuals, irrespective of the level of daily PA. Moreover, a significant predictive relationship was found between anticipatory pleasure and consummatory pleasure in the whole sample. Our findings build on previous research highlighting hedonic deficits in depression, showing that measures of state rather than trait anticipatory and consummatory pleasure provide better estimates for hedonic processing function in dysphoria. These findings contribute to the ecological validation of the two dimension construct of anhedonia and provide specific targets that can be used to refine existing therapeutic interventions.

## Data Availability

The datasets generated for this study are available on request to the corresponding author.

## Ethics Statement

The studies involving human participants were reviewed and approved by the Ethics Committee of the Central China Normal University. The patients/participants provided their written informed consent to participate in this study.

## Author Contributions

XL generated the idea, designed and supervised the study, and wrote the first draft of the manuscript. Y-TZ and Z-JH helped with data collection, data analysis, and manuscript writing. X-LC and F-HY helped with data collection and data analysis. X-JS commented significantly to the draft of the manuscript. All authors contributed to and have approved the final text.

## Conflict of Interest Statement

The authors declare that the research was conducted in the absence of any commercial or financial relationships that could be construed as a potential conflict of interest.
