# Supplementary material for: Diminished Anticipatory and Consummatory Pleasure in Dysphoria: Evidence From an Experience Sampling Study
Source: Front Psychol. 2019 Sep 19;10:2124. doi: 10.3389/fpsyg.2019.02124 (PMC6761272; doi:10.3389/fpsyg.2019.02124)
Supplement: Supplementary file 1 [file Table_1.DOCX]

**Supplementary materials**

Diminished anticipatory and consummatory pleasure in dysphoria: Evidence from an experience sampling study

Multilevel models were used to examine the moderating effect of dysphoric status on the association between consummatory pleasure*_ij_* and anticipatory pleasure *_t_*_-1_ (Model 3-1)_._ Additionally, consummatory pleasure at *t*-1 and positive affect (PA) at *t* were included as covariates separately (see Model 4-1, 5-1) and simultaneously (see Model 6-1) to control for potential confounding effects.

**Model 3-1**

Level 1: consummatory pleasure *_ij_* = ß_0_*_j_* + ß_1_*_j_* (anticipatory pleasure *_t_*_-1_) + r*_ij_*

Level 2: ß_0_*_j_* = γ_00_
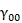
+ γ_01_ (dysphoric status *_j_*) + U_0_*_j_*
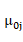


ß_1_*_j_* = γ_10_ + γ_11_ (dysphoric status *_j_*) + U_1_*_j_*
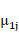


**Model 4-1**

Level 1: consummatory pleasure *_ij_* = ß_0_*_j_* + ß_1_*_j_* (anticipatory pleasure *_t_*_-1_) +

ß_2_*_j_* (consummatory pleasure *_t_*_-1_) + r*_ij_*

Level 2: ß_0_*_j_* = γ_00_ + γ_01_ (dysphoric status *_j_*) + U_0_*_j_*
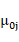


ß_1_*_j_* = γ_10_ + γ_11_ (dysphoric status *_j_*) + U_1_*_j_*

ß_2_*_j_* = γ_20_ + γ_21_ (dysphoric status *_j_*) + U_2_*_j_*
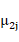


**Model 5-1**

Level 1: consummatory pleasure *_ij_* = ß_0_*_j_* + ß_1_*_j_* (anticipatory pleasure *_t_*_-1_) +

ß_2_*_j_* (PA *_t_*) + r_ij_

Level 2: ß_0_*_j_* = γ_00_ + γ_01_ (dysphoric status *_j_*) + U_0_*_j_*
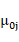


ß_1_*_j_* = γ_10_ + γ_11_ (dysphoric status *_j_*) + U_1_*_j_*
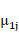


ß_2_*_j_* = γ_20_ + γ_21_ (dysphoric status *_j_*) + U_2_*_j_*
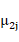


**Model 6-1**

Level 1: consummatory pleasure *_ij_* = ß_0_*_j_* + ß_1_*_j_* (anticipatory pleasure *_t_*_-1_) +

ß_2_*_j_* (consummatory pleasure *_t_*_-1_) + ß_3_*_j_* (PA *_t_*) + r*_ij_*

Level 2: ß_0_*_j_* = γ_00_ + γ_01_ (dysphoric status *_j_*) + U_0_*_j_*
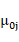


ß_1_*_j_* = γ_10_ + γ_11_ (dysphoric status *_j_*) + U_1_*_j_*

ß_2_*_j_* = γ_20_ + γ_21_ (dysphoric status *_j_*) + U_2_*_j_*
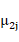


ß_3_*_j_* = γ_30_ + γ_31_ (dysphoric status *_j_*) + U_3_*_j_*
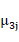


These models is highly similar to Model 3-6 with the exception that, dysphoric status (i.e., 0 = non-dysphoric group; 1 = dysphoric group) was added as a dummy variable at Level 2 to predict between-person variations of Level 1 parameters (intercept and slopes).

**Supplementary Table 1** The moderating effects of dysphoria status on the time-lagged associations between consummatory pleasure, anticipatory pleasure and positive affect (PA)

| Outcome variable:  Consummatory pleasure (*t*) | Predictors | *Est. diff (SE)* | *p* |
| --- | --- | --- | --- |
| **Model 3-1** | | |  |
|  | Anticipatory pleasure (*t*-1) | <-0.01 (0.05) | 0.94 |
| **Model 4-1** | | |  |
|  | Anticipatory pleasure (*t*-1) | -0.02 (0.05) | 0.66 |
|  | consummatory pleasure (*t*-1) | 0.03 (0.05) | 0.60 |
| **Model 5-1** | | |  |
|  | Anticipatory pleasure (*t*-1) | 0.03 (0.03) | 0.35 |
|  | PA (*t*) | 0.43 (0.23) | 0.06 |
| **Model 6-1** | | |  |
|  | Anticipatory pleasure (*t*-1) | 0.01 (0.04) | 0.79 |
|  | consummatory pleasure (*t*-1) | 0.02 (0.04) | 0.55 |
|  | PA (*t*) | 0.28 (0.21) | 0.19 |

Notes: *Est. diff* = differences of the estimated means between the dysphoric and non-dysphoric groups.
